# Hemoglobin–Albumin–Lymphocyte–Platelet (HALP) Score as a Predictive Model for the Success of Reconstruction of Head and Neck Defects with Free Microvascular Flaps

**DOI:** 10.3390/jcm12165314

**Published:** 2023-08-15

**Authors:** Marko Tarle, Igor Čvrljević, Marina Raguž, Ivica Lukšić

**Affiliations:** 1 Department of Maxillofacial Surgery, Dubrava University Hospital, 10000 Zagreb, Croatia; tarlemarko1@gmail.com (M.T.); igor.cvrljevic@gmail.com (I.Č.); 2 School of Dental Medicine, University of Zagreb, 10000 Zagreb, Croatia; 3 Department of Neurosurgery, Dubrava University Hospital, 10000 Zagreb, Croatia; marinaraguz@gmail.com; 4 School of Medicine, Catholic University of Croatia, 10000 Zagreb, Croatia; 5 School of Medicine, University of Zagreb, 10000 Zagreb, Croatia

**Keywords:** free flap, head and neck, HALP score, flap survival, microvascular surgery

## Abstract

Significant advances in reconstructive head and neck surgery with free microvascular flaps have had a positive impact on esthetic outcomes and quality of life. However, complications still occur in some patients. This study investigated the influence of the Hemoglobin, Albumin, Lymphocyte, and Platelet Score (HALP score), an immunonutritive marker, on complications and flap success. The retrospective analysis included 194 patients who underwent reconstruction of head and neck defects with free microvascular flaps. The HALP score correlated strongly with overall complications, including flap necrosis, infection, fistula, and hematoma. Hemoglobin, albumin, lymphocytes, and platelets individually showed associations with specific complications. HALP score was an extremely strong predictor of complications (AUC = 0.85). HALP score may be valuable for assessing patient status and predicting complications in microvascular free-flap reconstruction to allow timely interventions and improve outcomes. Further research is needed to investigate additional predictors and improve postoperative care.

## 1. Introduction

Significant advances have been made in head and neck reconstructive surgery with the use of free microvascular flaps, which have completely changed the surgical treatment paradigm for these patients and have had a significant positive impact on esthetic outcomes and quality of life [1]. In the mid-20th century, microvascular anastomosis techniques were developed that allowed for more precise reconstruction of head and neck defects [2]. The success rate of free-flap reconstruction ranges from 95% to 98%, but certain risks such as infection, hematoma, anastomotic insufficiency, and partial or complete flap necrosis still occur in 25% to 33% of patients [3,4,5,6]. These risks depend on several factors, including surgical technique, vascular status, patient health, and individual factors such as immune and nutritional status [7,8]. It is important that an experienced microvascular surgeon selects appropriate patients for free-flap reconstruction and chooses the appropriate flap based on the defect size and tissue type to be replaced. The most commonly used flaps for reconstruction of head and neck defects include the radial forearm flap, fibular flap, anterolateral thigh flap (ALT), deep circumflex iliac artery (DCIA) bone flap, and scapular flap [1]. The aim of this study is to analyze how the preoperatively determined Hemoglobin, Albumin, Lymphocyte, and Platelet Score (HALP score), an immunonutritive marker, influences the occurrence of complications and the success of the free flap itself. The HALP score is used to assess the nutritional status and overall health of the patient and is considered a prognostic factor in many malignancies, with lower HALP scores associated with poorer overall survival in cancer patients [9]. However, the prognostic value of the HALP score for outcomes in free microvascular flaps has not been studied.

## 2. Materials and Methods

This is a retrospective study of 194 patients who underwent surgery at the Department of Maxillofacial Surgery, National Center for Treatment of Tumors of the Face, Jaws and Mouth, Dubrava University Hospital, between 1 January 2012, and 31 December 2021. All participants underwent defect reconstruction in the head and neck region with free microvascular flaps. Microvascular reconstruction was performed by a team of maxillofacial surgeons who specialize in plastic and reconstructive surgery of the head and neck and have many years of clinical experience. Mostly, these were head and neck tumor surgeries performed in two surgical teams of maxillofacial surgeons who simultaneously performed resection of the primary tumor, raising of the free flap, and subsequent reconstruction of the defect with a microvascular flap. The majority of patients were oncological patients with head and neck malignant tumor. Preoperative laboratory tests were performed in all patients, and the concentrations of hemoglobin, albumin, lymphocytes, and platelets were used to calculate the HALP score according to the formula established by Chen et al.: Hemoglobin (g/L) × Albumin (g/L) × Lymphocytes (/L)/Platelets (/L) [10]. In addition, the following parameters were analyzed: age, sex, comorbidities, length of hospital stay, location of the defect, type of flap (radial forearm flap, fibular flap, anterolateral thigh flap (ALT), deep circumflex iliac artery (DCIA) bone flap, scapular flap, latissimus dorsi flap, rectus muscle flap, deep inferior epigastric perforator flap (DIEP), and serratus muscle flap), values of preoperative laboratory tests used to calculate the HALP score (hemoglobin, albumin, lymphocytes, and platelets) and complications of microvascular reconstruction (overall complications, flap revision, flap necrosis, infection, fistula, hematoma, partial flap necrosis). In the postoperative period, the flap was monitored for 72 h, including flap assessment and viability verification. To be included in this study, patients had to meet the following criteria: (a) presence of an indication for microvascular reconstruction of head and neck defects by a multidisciplinary team, (b) preoperative values of laboratory parameters required for the calculation of the HALP score, (c) fulfilled radiological and clinical criteria for the patient’s suitability for microvascular reconstruction (CT angiography and/or Doppler ultrasound examination of the flap area, Allen test, preoperative general medical assessment).

The exclusion criteria were as follows: (a) insufficient or conflicting data, (b) previous radiation therapy in the recipient and donor area, (c) previous chemotherapy, (d) previous major surgical procedures in the patient’s head and neck region, (e) previous trauma in the recipient area, (f) severe peripheral vascular disease, (g) lack of availability of adequate host vessels, (h) history of coagulopathy, (i) vasculitis, (j) connective tissue disease, (k) severe obesity, and (l) venous insufficiency. Medical records from the hospital information system were available for all participants.

This study was carried out in accordance with the recommendations of the ethics board of the Dubrava University Hospital. Written informed consent was obtained for all patients in accordance with the Declaration of Helsinki. The protocol was approved by the Institutional Review Board of the Dubrava University Hospital, Zagreb, Croatia (2023/2006-01, 20 June 2023).

### Statistical Analysis

Data analysis was performed with MedCalc Statistical Software version 12.5.0 (MedCalc Software, Ostend, Belgium; https://www.medcalc.org, accessed on 14 August 2022). Data were plotted as individual values, horizontal lines and markers represent mean  ±  SD. The distribution was assessed with the Kolmogorov–Smirnov test. The chi-square test and Fisher’s exact test were used for qualitative variables. Associations between complications related to the flap and other various patient data were assessed with the Pearson correlation coefficient, Spearman’s rank correlation, Point-Biserial correlation and phi coefficient; the level of corelation was set up as 0.3. In addition, logistic regression was used. The potential value for predicting the occurrence of complications was assessed using receiver operating characteristic (ROC) curve analysis. Statistical significance was set at *p* < 0.05.

## 3. Results

Between 1 January 2012 and 31 December 2021, a total of 194 consecutive patients (134 males, 69.1% and 60 females, 30.9%) were enrolled in the study. Average age of included patients was 60 ± 12.54 years (Table 1).

Complications occurred overall in 29.4% of patients (57/194); hematoma in 5.7% (11/194), infection in 14.9% (29/194), fistula in 10.3% (20/194), necrosis in 8.2% (16/194), and partial necrosis in 4.1% of patients (8/194). Revision of microvascular flap was performed in 11.2% of patients (22/194) (Table 2). The majority of patients had no concomitant diseases (59%), while the rest suffered mainly from arterial hypertension and diabetes.

The average HALP value in the presented study was 30.31 ± 19.16, arithmetic mean 34.22 with 95% CI 31.50 to 36.93.

Hemoglobin (r_pb_ = −0.26, *p* = 0.00025), albumin (r_pb_ = −0.46, *p* < 0.00001), lymphocytes (r_pb_ = −0.26, *p* = 0.00027), platelets (r_pb_ = 0.27, *p* = 0.00012), as well as HALP score (r_pb_ = −0.48, <0.00001) showed a strong association with the occurrence of complications overall. Moreover, hemoglobin showed an association with the occurrence of flap necrosis (r_pb_ = −0.17, *p* = 0.02), infection (r_pb_ = −0.15, *p* = 0.04), and flap revision (r_pb_ = −0.23, *p* = 0.0018); albumin with the occurrence of flap necrosis (r_pb_ = −0.41, *p* = 0.00001), infection (r_pb_ = −0.28, *p* = 0.00006), fistula (r_pb_ = −0.18, *p* = 0.010), hematoma (r_pb_ = −0.18, *p* = 0.011), and flap revision (r_pb_ = −0.39, *p* < 0.00001); lymphocytes with the occurrence of hematoma (r_pb_ = −0.17, *p* = 0.024), and partial necrosis (r_pb_ = −0.16, *p* = 0.029), while platelets showed an association with the occurrence of infection (r_pb_ = 0.34, *p* < 0.00001), and fistula (r_pb_ = 0.13, *p* = 0.05). Interestingly, HALP score showed a strong association with the occurrence of flap necrosis (r_pb_ = −0.23, *p* = 0.0015), infection (r_pb_ = −0.35, *p* < 0.00001), fistula (r_pb_ = −0.26, *p* = 0.0003), hematoma (r_pb_ = −0.14, *p* = 0.05), partial flap necrosis (r_pb_ = −0.16, *p* = 0.024), as well as flap revision (r_pb_ = −0.26, *p* < 0.00019).

Hospital stays showed a strong association with occurrence of complication (r_pb_ = 0.33, *p* < 0.00001), especially necrosis (r_pb_ = 0.44, *p* < 0.00001), infection (r_pb_ = 0.28, *p* = 0.00008), fistula (r_pb_ = 0.30, *p* = 0.00003), and albumin levels (r = −0.21, *p* = 0.004). In addition, tumor localization showed association with complication occurrence (r_pb_ = 0.15, *p* = 0.035), while type of flap was not associated with complication (r_pb_ = 0.09, *p* = 0.210). The correlation between the presence of certain comorbidities and various clinical parameters, complications related to microvascular reconstruction and the HALP score, and the individual laboratory parameters analyzed were not statistically significant.

**Figure 1:**
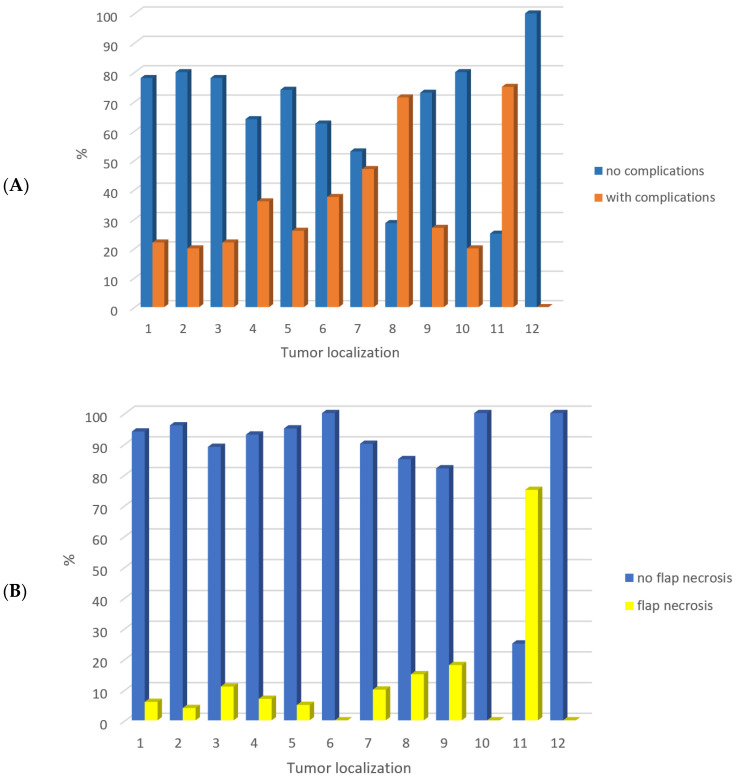

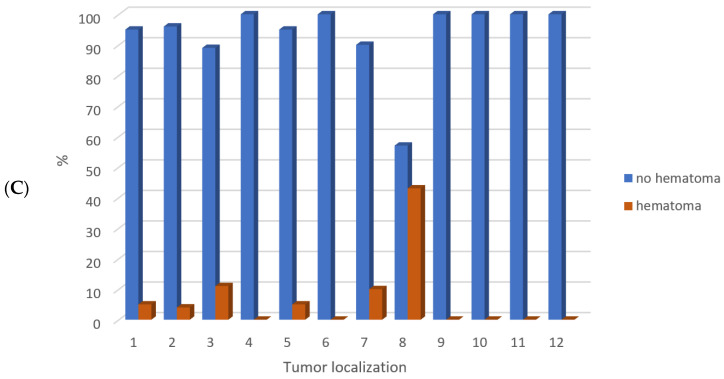
The frequency of head and neck tumor localization and overall complication occurrence (*p* = 0.07, Fisher’s exact test) (**A**), flap necrosis (*p* = 0.03, Fisher’s exact test) (**B**), and hematoma (*p* = 0.05, Fisher’s exact test) (**C**). Legend: 1 gingiva of the mandibula 46/194 (23.7%), 2 tongue 25/194 (12.9%), 3 cheek 9/194 (4.6%), 4 retromolar 14/194 (7.2%), 5 floor of the oral cavity 43/194 (22.2%), 6 oropharynx 8/194 (4.1%), 7 maxilla 19/194 (9.8%), 8 nasal cavity 7/194 (3.6%), 9 skin of the face 11/194 (5.7%), 10 palate 5/194 (2.6%), 11 skull base 4/194 (2.1%), 12 orbit 3/194 (1.5%).

We analyzed the frequency of head and neck tumor localization and overall complication occurrence (*p* = 0.07, Fisher’s exact test), as well as frequency of tumor localization and necrosis (*p* = 0.03, Fisher’s exact test), and hematoma (*p* = 0.05, Fisher’s exact test) (Figure 1).

**Figure 2:**
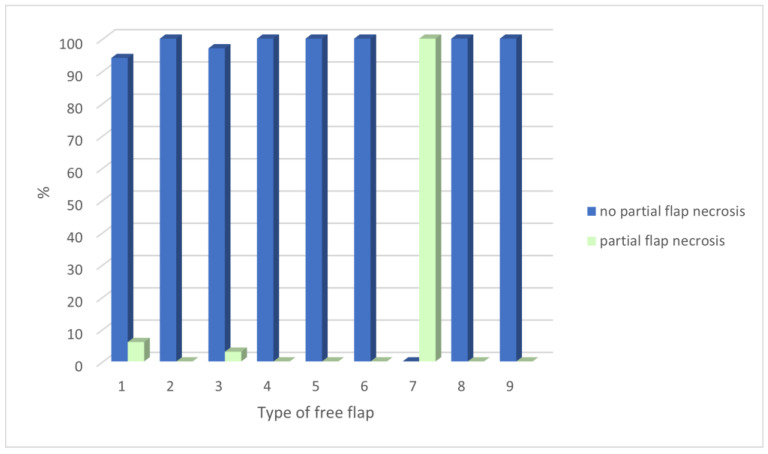
The frequency of type of microvascular flap and partial necrosis complication (*p* = 0.07, Fisher’s exact test). Legend: Type of microvascular flap: 1—forearm 95/194 (49.0%), 2—fibula 47/194 (24.2%), 3—ALT 37/194 (19.1%), 4—DCIA 9/194 (4.6%), 5—scapular 1/194 (0.5%), 6—latisimus 2/194 (1.0%), 7—DIEP 1/194 (0.5%), 8—rectus abdominis 1/194 (0.5%) and 9—serratus flap 1/194 (0.5%).

Furthermore, we analyzed the type of microvascular flap and complication occurrence, especially necrosis (*p* = 0.04, Fisher’s exact test), as well as partial necrosis (*p* = 0.07, Fisher’s exact test) (Figure 2).

**Figure 3:**
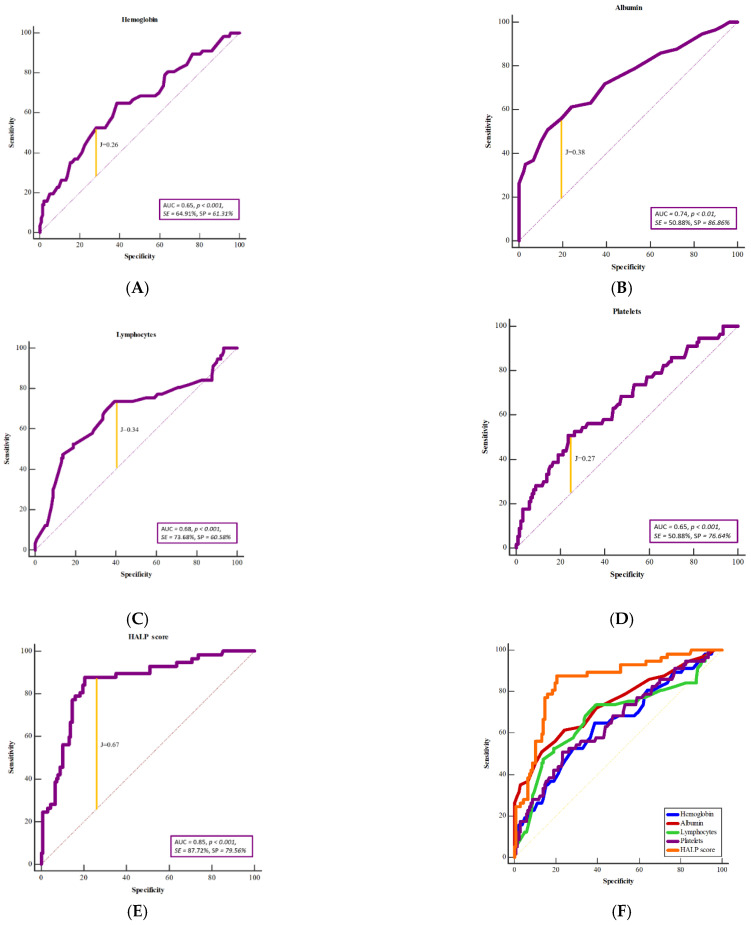
Receiver operating characteristic curve analysis (ROC) for overall complication to show the potential prognostic value of hemoglobin level (**A**), platelet level (**B**), albumin level (**C**), lymphocyte level (**D**), and HALP score (**E**). The HALP score has the greatest prognostic significance in predicting overall complications associated with the microvascular flap compared with the individual laboratory parameters (**F**). AUC—area under the curve, SE—sensitivity (%), SP—specificity (%), J—Youden index.

To determine the value of clinical parameters (hemoglobin, albumin, lymphocytes, platelets, and HALP score) in predicting potential complications, an ROC analysis was performed. The level of hemoglobin (area under the curve, AUC = 0.65; *p* < 0.001, sensitivity, SE = 64.91%, specificity, SP = 61.31%, Youden index, J = 0.26, positive predictive value, PPV = 41.10%, negative predictive value, NPV = 80.76%), albumin (AUC = 0.74; *p* < 0.001, SE = 50.88%, SP = 86.86%, J = 0.38, PPV = 61.69%, NPV = 80.95%), lymphocytes (AUC = 0.68; *p* < 0.001, SE = 73.68%, SP = 60.58%, J = 0.34, PPV = 43.74%, NPV = 84.69%), and platelets (AUC = 0.65; *p* < 0.001, SE = 50.88%, SP = 76.64%, J = 0.27, PPV = 47.53%, NPV = 78.94%) are moderate indicators of possible complications, while HALP score represents a strong indicator of possible complications (AUC = 0.85; *p* < 0.001, SE = 87.72%, SP = 79.56%, J = 0.67, PPV = 64.01%, NPV = 93.96%) (Figure 3).

Additionally, HALP score represents a moderate to strong indicator for occurrence of necrosis (AUC = 0.77; *p* < 0.001, SE = 81.82%, SP = 67.42%, J = 0.48, PPV = 18.25%, NPV = 97.56%), infection (AUC = 0.82; *p* < 0.001, SE = 93.10%, SP = 69.09%, J = 0.62, PPV = 34.61%, NPV = 98.27%), fistula (AUC = 0.78; *p* < 0.001, SE = 90.00%, SP = 66.09%, J = 0.56, PPV = 23.37%, NPV = 98.29%), hematoma (AUC = 0.68; *p* = 0.027, SE = 72.73%, SP = 69.95%, J = 0.42, PPV = 12.70%, NPV = 97.71%), partial necrosis (AUC = 0.77; *p* < 0.001, SE = 87.50%, SP = 62.37%, J = 0.49, PPV = 9.08%, NPV = 99.14%), and flap revision (AUC = 0.78; *p* < 0.0001, SE = 81.82%, SP = 69.19%, J = 0.51, PPV = 25.35%, NPV = 96.74%).

**Figure 4:**
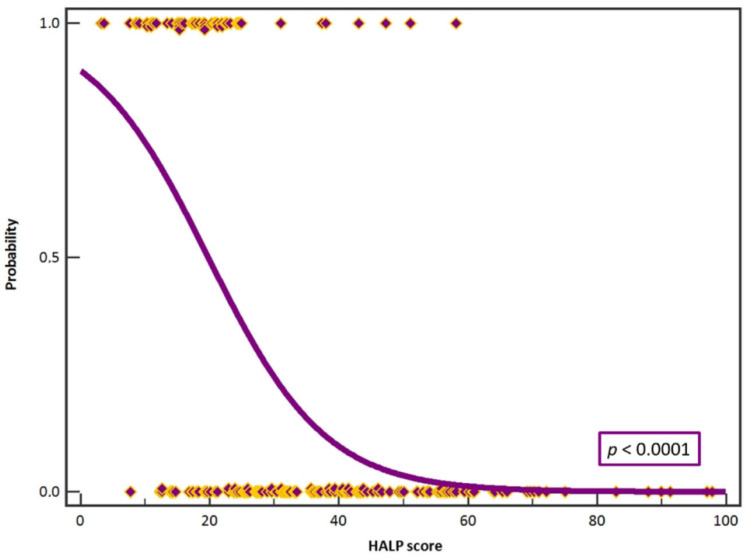
Logistic regression analysis showed statistically significant association of preoperative HALP value with occurrence of complications (*p* < 0.0001, R2 = 0.40). Purple boxes with yellow outlines present cases.

Linear regression analysis showed statistically significant association of preoperative HALP value with occurrence of complications (ρ = −0.55, *p* < 0.0001, r = −0.55, R2 = 0.23) (Figure 4).

**Figure 5:**
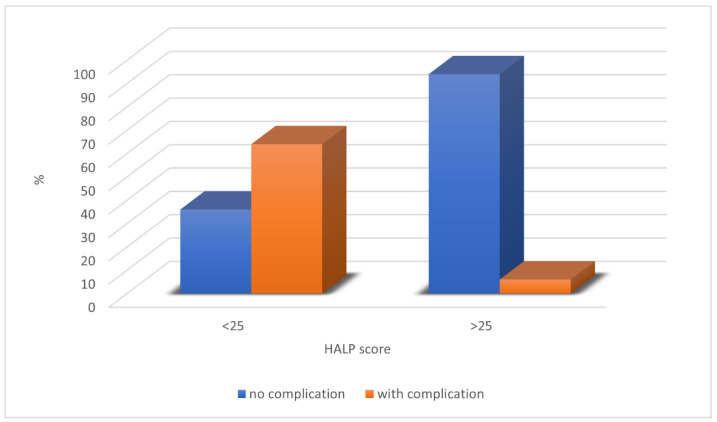
The HALP score cut-off value of 25 can be used as a reference value for predicting the occurrence of complications before surgery in patients requiring a reconstructive procedure with a free microvascular flap.

Furthermore, we set up the HALP score cut-off value of 25. Statistical significance was shown between the groups of subjects (χ^2^ = 75.41, *p* < 0.0001). According to our analysis, a HALP score of 25 can be used as a reference value for predicting the occurrence of complications before surgery in patients requiring a reconstructive procedure with a free microvascular flap (Figure 5).

## 4. Discussion

In this study, we investigated the prognostic impact of the immunonutritive marker, HALP score, on the occurrence of complications and survival of free microvascular flaps used in reconstruction of head and neck defects. The HALP score, described in 2015 by Chen et al., integrates indicators of immune status (lymphocyte and platelet counts) and nutritional status (hemoglobin and albumin concentrations) that are routinely assessed with basic laboratory tests [10]. Previous research suggests that lower HALP levels may be associated with poorer prognostic outcomes in various malignancies [9]. In 2023, Xu et al. performed a meta-analysis involving 13,110 cancer patients and demonstrated that a low HALP score is associated with shortened overall survival (OS), progression-free survival (PFS), disease-free survival (DFS), and recurrence-free survival (RFS), making it a reliable negative prognostic biomarker for cancer patient survival [11]. According to the available literature, no study has specifically investigated the impact of HALP score values on free microvascular flap success.

This study analyzed the preoperative laboratory parameters used to calculate the HALP score in 194 subjects who underwent reconstruction of defects in the head and neck region with a free microvascular flap. The forearm flap (95/194), fibular flap (47/194), and ALT flap (37/194) were the most commonly used flaps in this study, consistent with the literature [1,12]. Complications related to the free flap occurred in 29.4% of subjects, the most common of which were infection (14.9%) and the need for flap revision (11.2%) due to arterial or venous problems with the anastomosis. The number of complications is consistent with what is reported in the literature [3,4,5,6]. Flap necrosis requiring repeat reconstructive surgery occurred in only 8.2% of patients. Although the aforementioned patients were treated preoperatively and were suitable candidates for microvascular reconstruction according to clinical and radiological criteria, complications occurred in one third of patients, making it necessary to find new risk factors and indicators of unfavorable outcomes of microvascular reconstruction [13,14,15].

Nutritional status of surgical patients is an important factor in the occurrence of postoperative complications. Adequate protein levels are required for wound healing and immune response [7]. Albumin has several vital physiological functions, including maintenance of microvascular integrity and colloid osmotic pressure, and it plays a role in anticoagulation, antioxidant processes, and detoxification. Low albumin levels have a negative effect on wound healing because albumin promotes collagen synthesis and granuloma formation. In addition, hypoalbuminemia impairs the immune response. Low preoperative albumin concentrations are associated with poor patient nutritional status and may affect survival of the microvascular flap itself [16,17]. Because most of our patients had advanced-stage malignant head and neck tumor, many of them had low albumin levels preoperatively. Shum demonstrated in 162 patients that patients with low albumin levels before reconstructive head and neck surgery had a four-fold higher risk of microvascular free-flap failure than patients with normal albumin levels [18]. Xu found in 315 patients with head and neck tumors that perioperative serum albumin supplementation positively influenced the occurrence of surgery-related local complications (6.5% vs. 21.6%) associated with head and neck free flaps, as well as length of hospital stay [19]. Our results are consistent with the above. Patients with complications related to the microvascular flap were more likely to have decreased albumin levels preoperatively. These patients had higher rates of flap revision, infection, necrosis, and fistula. In addition, decreased albumin levels were found to be a moderately strong predictor of complications (AUC = 0.74; *p* < 0.001, SE = 50.88%, SP = 86.86%).

Although anemia may have a positive effect on free flap perfusion because of decreased blood viscosity, resulting in decreased resistance in blood vessels and increased cardiac output, an increase in Reynolds number or turbulent blood flow increases the risk of thrombosis. In addition, loss of sympathetic function in the arterioles of the free flap leads to vasodilation, which decreases the velocity of blood flow and promotes thrombogenesis. In addition, capillary vasoconstriction in the flap resulting from the release of vasodilators from the endothelium, together with decreased shear stress in response to decreased blood viscosity without a concomitant increase in blood flow, theoretically leads to decreased oxygenation of the flap [20,21]. Preoperative low hemoglobin levels are significant predictors of free-flap failure. Hill and colleagues demonstrated in 147 patients that low hemoglobin and hematocrit levels negatively affect free-flap survival [22]. Our study showed a statistically significant negative association between hemoglobin level and all complications, need for flap revision, and flap necrosis, which is consistent with data from the literature.

Lymphocytes play a critical role in regulating the immune response by recruiting and activating various inflammatory mediators and transcription factors and promoting systemic inflammatory responses, thus playing an important role in protecting against infection [23]. Yu examined the effects of prognostic nutrition index (PNI) values on free-flap survival. The PNI is defined as the product of albumin and lymphocyte levels. Lower PNI values were found to be associated with free-flap failure and longer hospital stay [7]. Complications, especially the occurrence of fistulas, hematomas, and partial necrosis of the flap, correlate negatively with lymphocyte levels in our study. Decreased lymphocyte levels are a moderately negative prognostic factor for complications (AUC = 0.68; *p* < 0.001, SE = 73.68%, SP = 60.58%).

Thrombocytosis may lead to circulatory disturbances in the distal portions of the free flap due to microthrombus formation. In reactive and primary thrombocytosis, elevated platelet counts increase the risk of venous thrombosis. The release of vasoconstrictive substances from platelets may affect the capillary network of the free flap and cause ischemia [24]. Elevated preoperative platelet levels (>300 × 10^9^/L) are associated with an increased risk of free-flap failure and reoperation [25]. Because most of our patients had malignant tumors, thrombocytosis and hypercoagulable states can be explained in the context of a paraneoplastic syndrome. A cohort study of 3744 patients undergoing reconstructive surgery with microvascular flaps demonstrated an association between thrombocytosis and increased risk of complications, including the need for blood transfusions, prolonged hospital stays, and reoperation [25]. In contrast to all observed laboratory parameters, we demonstrated a positive correlation between the value of platelets (indicator of immune status) and the occurrence of complications, especially infections and fistulas, indicating the correctness of using the HALP score in evaluating the risk of a poor outcome of microvascular reconstruction.

Although all observed laboratory parameters proved to be moderate predictors of complications, the HALP score proved to be an extremely strong predictor (AUC = 0.85; *p* < 0.001, SE = 87.72%, SP = 79.56%) of overall complications as a moderate to strong indicator of the occurrence of four of the five complications (necrosis, infection, fistula, partial necrosis, and flap revision). These facts justify the use of the HALP score as a predictor of free-flap failure. Because no such study has been performed to date, we determined the cut-off value of the HALP score. A HALP score less than 25 was associated with the occurrence of complications in the postoperative period. This suggests that the combination of reduced hemoglobin, lymphocyte, or albumin levels or increased platelet levels may adversely affect the outcome of microvascular reconstruction.

In addition, we have demonstrated a statistically significant positive correlation between almost all complications and length of hospital stay. It is clear that the occurrence of a complication requires additional treatment and prolongs hospital stay, which explains these results. Of the laboratory parameters observed, a decreased albumin level was influenced by a prolonged hospital stay, which is in agreement with numerous studies [16,17,19].

Our study potentially offers new insights into the risk assessment of postoperative complications after microvascular reconstruction of head and neck defects; however, the presented results should be considered with several limitations. First, the retrospective nature of the study and the experience of a single center are the main drawbacks of the study. In addition, because of a lack of data, we could not follow the dynamics of the HALP score postoperatively, which would have allowed an even more precise statement about the importance of determining this score. Finally, we cannot exclude other potential factors that may lead to complications related to microvascular reconstruction.

## 5. Conclusions

The results of our study suggest that the HALP score, as a useful tool, can provide relevant information about the immunonutritive status of patients and predict complications and outcomes in successful reconstruction of microvascular free flaps in the head and neck region. Identifying patients with low HALP scores (<25) prior to surgery will allow us to intervene in a timely manner and prepare the patient for reconstructive surgery, which will impact the patient’s microvascular reconstruction outcomes and hospital stay. Further studies are needed to confirm our findings and to investigate other potential predictors of microvascular reconstruction success. Understanding the association between the HALP score and the success of microvascular flap reconstruction may help improve postoperative care and optimize the outcomes of this complex surgical procedure. It is important to note that the HALP score is not the only factor influencing free-flap success, and other risk factors must be considered when deciding on a free microvascular flap.

## Figures and Tables

**Table 1:** Demographic and clinical data of the subjects.

	Total	Males	Females
Number of patients	194	134	60
Age (years)	60 ± 12.54	60 ± 11.46	60.41 ± 14.78
Hospital stay (days)	33.41 ± 18.41	33.64 ± 17.90	32.90 ± 19.66
Number of overall flap complication	57	39	18

**Table 2:** Distribution of total and individual complications in microvascular reconstructions depending on the type of free flap.

Type of Free Flap	Number of Free Flaps	Overall Complications	Flap Revision	Necrosis	Infection	Fistula	Hematoma	Partial Necrosis
Radial forearm flap	95	26	9	5	14	10	6	6
Fibular flap	47	12	6	4	5	4	3	0
ALT flap	37	14	5	4	7	5	2	1
DCIA flap	9	2	1	1	1	0	0	0
Scapular flap	1	0	0	0	0	0	0	0
Latissimus dorsi flap	2	1	1	1	0	0	0	0
DIEP flap	1	1	0	0	1	0	0	1
Rectus muscle flap	1	1	0	1	1	1	0	0
Serratus muscle flap	1	0	0	0	0	0	0	0
Total	194	57 (29.4%)	22 (11.2%)	16 (8.2%)	29 (14.9%)	20 (10.3%)	11 (5.7%)	8 (4.1%)

## Data Availability

All data generated or analyzed during this study are included in this published article.
